# Apolipoprotein D expression does not predict breast cancer recurrence among tamoxifen-treated patients

**DOI:** 10.1371/journal.pone.0171453

**Published:** 2017-03-16

**Authors:** Daniella Klebaner, Stephen Hamilton-Dutoit, Thomas Ahern, Anatasha Crawford, Thomas Jakobsen, Deirdre P. Cronin-Fenton, Per Damkier, Emiel Janssen, Anders Kjaersgaard, Anne Gulbech Ording, Håvard Søiland, Henrik Toft Sørensen, Timothy L. Lash, Ylva Hellberg

**Affiliations:** 1 Department of Epidemiology, Rollins School of Public Health, Emory University, Atlanta, Georgia, United States of America; 2 Institute of Pathology, Aarhus University Hospital, Aarhus, Denmark; 3 Departments of Surgery and Biochemistry, University of Vermont College of Medicine, Burlington, Vermont, United States of America; 4 Department of Clinical Epidemiology, Aarhus University, Aarhus, Denmark; 5 Department of Clinical Chemistry & Pharmacology, Odense University Hospital, Odense, Denmark; 6 Department of Pathology, Stavanger University Hospital, Stavanger, Norway; 7 Department of Breast and Endocrine Surgery, Stavanger University Hospital, Stavanger, Norway; 8 Department of Clinical Science, University of Bergen, Bergen, Norway; Turun Yliopisto, FINLAND

## Abstract

**Background:**

Apolipoprotein D (ApoD) has been proposed as a predictor of breast cancer recurrence among estrogen receptor-positive (ER+), tamoxifen-treated patients.

**Methods:**

We conducted a population-based case-control study nested in a population of 11,251 women aged 35–69 years at diagnosis with Stage I–III breast cancer between 1985 and 2001 on Denmark’s Jutland Peninsula and registered with the Danish Breast Cancer Cooperative Group. We identified 541 recurrent or contralateral breast cancers cases among women with ER+ disease treated with tamoxifen for at least 1 year and 300 cases in women with ER– disease never treated with tamoxifen. We matched one control subject per case and assessed ApoD expression in the tumor cell nucleus and cytoplasm using tissue microarray immunohistochemistry. We computed the odds ratio (OR) associating ApoD expression with recurrence and adjusted for potential confounding using logistic regression.

**Results:**

Cytoplasmic ApoD expression was seen in 68% of ER+ tumors, in 66% of ER– tumors, and in 66% of controls across both groups. In women with ER+ tumors, the associations of cytoplasmic ApoD expression with recurrence (OR = 1.0; 95% CI = 0.7 to 1.4) and increasing cytoplasmic expression with recurrence (OR = 1.0; 95% CI = 0.996 to 1.003) were null, as were those for women with ER– tumors. Associations for nuclear ApoD expression and combined nuclear and cytoplasmic expression were similarly near-null.

**Conclusion:**

ApoD expression is likely not a predictor of recurrence in tamoxifen-treated patients.

**Impact:**

This study eliminates the previously suggested marker ApoD as a predictor of recurrence among tamoxifen-treated women.

## Introduction

Breast cancer accounts for the highest number of cancer cases among women worldwide, and is the second leading cause of cancer death.[1, 2] Between two-thirds and three-quarters of breast tumors express the estrogen-receptor-alpha (ERα) protein.[3, 4] Patients with ER+ breast cancers usually receive adjuvant anti-estrogen therapy, typically tamoxifen—a selective ER modulator—or an aromatase inhibitor. [5–11] Tamoxifen selectively binds to the ligand-binding domain of the ER, blocking estrogen’s ability to bind and induce proliferation of the cancer cells.[12–14]

In spite of tamoxifen’s measurable positive effect on breast cancer prognosis, only about 70% of all ER+ breast cancers respond to anti-estrogen therapies.[12, 15] In addition, many breast cancers that initially respond eventually develop resistance to these therapies.[16] Effective use of anti-estrogen therapy may depend on the ability to subtype receptor-positive breast cancers based on their biomarker profiles.[15, 17–19] Despite a number of studies on the subject, to date, no biomarker has been translated into clinical practice to predict which tamoxifen-treated patients are at high risk for recurrence.[20]

Apolipoprotein D (ApoD) expression may be predictive of recurrence among tamoxifen-treated patients.[21–24] ApoD is a 29-kDa glycoprotein involved in transport of hydrophobic ligands and is ubiquitous in human tissue.[25–27] Molecular studies have demonstrated an inhibitory effect of the ER on ApoD, with up-regulation following tamoxifen treatment, likely through blockage of ER activity.[28] As a result, combined ER positivity and ApoD positivity could reflect a malfunctioning hormone receptor pathway, resulting in ineffective tamoxifen treatment and a higher risk for relapse.[27–31] Only two studies have explored the direct relation between ApoD expression and recurrence among tamoxifen-treated patients.[26, 32] We sought to examine precisely this association in a larger, well-characterized population.

## Materials and methods

### Patients

Using the Danish Breast Cancer Cooperative Group (DBCG) database, we collected information on 11,251 female residents of the Jutland Peninsula between the ages of 35 and 69 who were diagnosed with Stage I–III invasive breast cancer between 1985 and 2001.[33, 34] The DBCG ends routine follow-up of breast cancer patients at ten years. For the purposes of this study, data were collected beginning at one year after the date of diagnosis, and ending on the date of a first breast cancer recurrence, death from any cause, loss to follow-up due to emigration, after ten years, or September 1, 2006 (the end of the study’s follow-up period) by linkage to the Civil Registration System.[35, 36] All data were linked using Danish Personal Registration Numbers.

Patients were divided into two subgroups—those whose tumors showed expression of ER and who had been on tamoxifen therapy for at least one year, and those whose tumors did not show expression of ER, were not treated with tamoxifen, and who had survived for at least one year. All other patients were excluded, including patients who received neoadjuvant treatment, though this was highly uncommon during the study period in Denmark.[37] Tumors were considered ER+ if ≥10% of cells expressed the ER.

The DBCG data provided recurrence information, defined as breast cancer—including contralateral cancer—or distant metastases diagnosed after receipt of initial treatment. Because tamoxifen reduces the rate of local, distant, and contralateral recurrences, these were grouped under a global heading of”recurrence” for assessing the direct predictive effect of ApoD on recurrence via the tamoxifen pathway. For the purposes of this study, a “case” was defined as a recurrence that occurred within ten years of the initial diagnosis. Controls were free of recurrence at the time of matching, and were matched to cases on ER status, menopausal status, stage, calendar time of diagnosis, and county.

Patients were also excluded if they had insufficient or invalid tissue material that could not be scored on the tissue microarrays (TMAs). Invalid tissue included tumors that were non-invasive, such as cores with only ductal carcinoma *in situ*, cores that were excessively over stained, cores that were damaged or cores that were missing from the stained sections. In total, 371 patients were excluded because there was insufficient valid tissue for ApoD determination. After these exclusions for quality control, 1,267 women remained in the study, each tumor being represented by between one and four evaluable TMA cores.

### Immunohistochemistry

Tissues were processed from formalin-fixed paraffin-embedded tissue blocks obtained from pathology department archives using sterile protocols designed to avoid cross-contamination. Cylindrical cores were sampled from each donor tumor and re-embedded into recipient TMA paraffin blocks using standard procedures. TMAs were constructed using a TMA Master (3DHistech Ltd., Budapest, Hungary) using 1 mm diameter cores. An asymmetrical design was employed, using liver and placental tissue tissue cores as TMA orientation markers and as controls. When possible, up to a maximum of three representative tumor cores and one marginal tissue core were sampled from each donor block (one block per patient), including a core with normal tissue for quality control.

We used immunohistochemistry to determine the subcellular expression intensity and location of ApoD. ApoD expression was detected using a rabbit monoclonal primary antibody at a 1:250 dilution, with thirty minutes of incubation time at room temperature (clone EPR2916, Abcam, Cambridge, UK). We performed heat induced epitope retrieval using a target retrieval solution with pH of 9. The visualization system used was Envision Flex, which uses 3,3’-diaminobenzidine as a chromogen and horseradish peroxidase as a labeled polymer. The stained TMA sections were scanned at 40x magnification with the Hamamatsu Nanozoomer 2.0HT in .ndpi format. Slides were converted with a beta version converter to conform to the 3DHistech software and uploaded to Panoramic Viewer TMA Module software.

### Scoring of TMA cores

Laboratory personnel, including scorers, were blinded to all clinical information, including case or control status, ER status, and receipt of therapeutic agents. The stained TMA breast carcinoma sections were read and scored for Apo-D expression by three observers (one experienced pathologist (YH), and two trained students (DK and TJ)). Initially, 62 cores were independently scored by the three raters, and agreement was compared for each rater pair. For ascertainment of positive cytoplasmic staining, agreement was generally good, with an average concordance of 89%. Concordance of positive nuclear staining was lower, with 79% agreement on average, although the total number of positively stained nuclei in the test sample was low, leading to less precise measures of agreement. The pathologist reviewed all the TMA cores after they were initially scored by the two other raters, to maximize scoring consistency. Discordantly scored cores were discussed by all three raters and a consensus score was agreed. Cores were excluded if they were excessively torn, over-stained, or consisted exclusively of carcinoma in situ or normal tissue. If a combination of normal tissue and tumor was present, only the portion with tumor was scored for ApoD.

### Definitions of analytical variables

#### ApoD cytoplasmic staining

**Fig 1:**
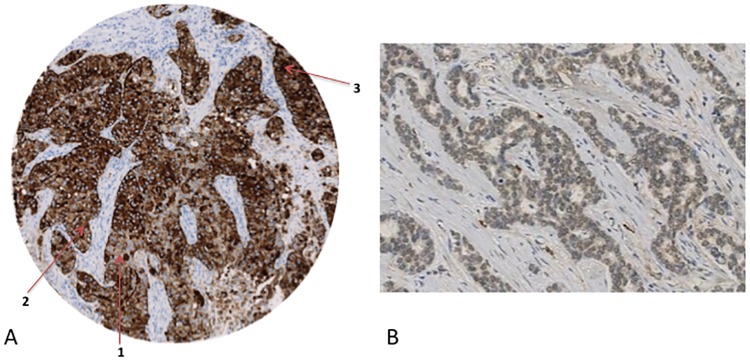
ApoD staining determinationζ §. §The source population consisted of 11,251 female residents of the Jutland Peninsula in Denmark aged 35–69 years who were diagnosed with Stage I, II, or III breast cancer between 1985 and 2001. ζ Brown signifies positive ApoD staining. A.Image depicts an immunohistochemically stained TMA core that was scored for cytoplasmic ApoD by the three raters as follows: 45% tumor cells weakly positive (1), 50% tumor cells moderately positive (2), and 5% tumor cells strongly positive (3). B. Image depicts a TMA core with widespread, weak nuclear staining.

A semi-quantitative scoring system employing a histological score, or “H-score,” was used for all analyses, as described by Soiland et al.[38] Briefly, the H-score weights the proportion of tumor cells in each TMA core by the intensity of their staining according to a four-level ordinal scale (0/negative, 1/weak, 2/moderate, or 3/strong). Soiland *et al*. identified a threshold H-score of 0 as a significant cutoff for patients over seventy years of age. Thus, patients with an H-score of zero were considered to have no ApoD expression, while patients with an H-score >0 were considered to have ApoD present. Two variables were used separately in the analysis—one dichotomous variable specifying staining as negative versus positive, and one continuous variable with the H-score itself (1–300) to assess dose-response (Fig 1a).

#### ApoD nuclear staining

Nuclear staining was quantified using a simplified nuclear N-score, defined as the product between intensity of staining (1/light, 2/dark) and frequency of staining (0/negative, 1/<25% positive nuclei, 2/25+ positive nuclei). Two variables were used for this analysis as with cytoplasmic staining—one dichotomous (negative versus positive, cutoff of 0), and one continuous (1–4) using the nuclear H-score (Fig 1b).

### Statistical analysis

All statistical analyses were conducted using SAS 9.3 (SAS Institute, Cary, NC). All analyses were performed within strata of ER+/TAM+ and ER–/TAM– to isolate the association between ApoD and recurrence. The ER–/TAM– group was included to examine any direct prognostic effect of ApoD expression on recurrence. Crude frequencies were calculated within the two strata showing the proportion of cases and controls in various categories of ApoD staining, separated into nuclear and cytoplasmic staining. Conditional logistic regression was used to calculate measures of association, with recurrence as the outcome and ApoD staining status as the exposure variable. Odds ratios estimating the association of ApoD positivity in the nucleus or cytoplasm with recurrence were computed, controlling for covariates. Matched odds ratios (OR) were compared with those computed with additional adjustment for receipt of chemotherapy, receipt of radiation therapy, and type of surgery. Within continuous categorization of ApoD staining status, the OR was computed excluding the negative referent group to assess the presence of a dose-response. We replicated analyses within strata of stage to further assess the predictive value of ApoD. A probabilistic bias analysis was performed to account for potential misclassification of ApoD staining, the results and methods of which are presented in S1 File and S1 Table.

This study was approved by the Danish Data Protection Agency (journal number 2012-41-1170) and the Ethical Committee (record number 1-10-72-16-15).

## Results

### Descriptive statistics

The majority of women were either Stage II (45%) or III (53%) at diagnosis, with only 2% designated as Stage I according to standards set by the Union for International Cancer Control.[34] Approximately half of the women were between the ages of 55–64, with 24% in the 65–69 category, 22% in the 45–54 category, and only 3% of women between the ages of 35 and 44 years. Given the age distribution, only 6% of women were premenopausal (Table 1).

**Table 1:** Frequency and proportion of breast cancer recurrence case patients and matched control subjects within group strata*.

Patient Characteristic	ER+/TAM+, No.(%)		ER-/TAM-, No.(%)	
	Recurrent Cases	Controls	Recurrent Cases	Controls
Cytoplasmic ApoD Expression (H-Score)				
= 0	135 (32)	144 (34)	80 (34)	77 (35)
>0	292 (68)	280 (66)	157 (66)	146 (66)
Missing †	114	117	63	77
Nuclear ApoD Expression (H-Score)				
= 0	260 (61)	258 (61)	169 (71)	165 (74)
>0	167 (39)	166 (39)	68 (29)	58 (26)
Missing †	114	117	63	77
Joint ApoD Expressionζ				
= 0	115 (44)	131 (46)	76 (54)	71 (58)
>0	147 (56)	153 (54	64 (46)	52 (42)
Missing †	279	257	160	177
Diagnosis Year§				
1985–1993	235 (43)	234 (43.3)	107 (36)	100 (33)
1994–1996	113 (21)	112 (20.7)	81 (27)	83 (28)
1997–2001	193 (36)	195 (36)	112 (37)	117 (39)
Age category at diagnosis, yrs				
35–44	13 (3)	12 (2.8)	52 (22)	41 (18)
45–54	92 (22)	86 (20)	94 (40)	84 (38)
55–64	221 (52)	222 (52)	67 (28)	68 (31)
65–69	101 (24)	104 (25)	24 (10)	30 (13)
Menopausal Status at diagnosis§				
Premenopausal	34 (6.3)	34 (6.3)	121 (40)	121 (40)
Postmenopausal	507 (94)	507 (94)	179 (60)	179 (60)
UICC tumor stage at diagnosis§				
I	8 (1.9)	6 (1.4)	14 (5.9)	15 (6.7)
II	194 (45)	193 (46)	128 (54)	115 (52)
III	225 (53)	225 (53)	95 (40)	93 (42)
Nodal involvement at diagnosis				
0	33 (6.1)	39 (7.2)	82 (27)	110 (37)
1–3	240 (44)	251 (46)	101 (34)	76 (25)
4–9	177 (33)	196 (36)	76 (25)	84 (28)
10+	91 (17)	55 (10)	41 (14)	30 (10)
Histological grade				
I	108 (20)	144 (27)	27 (9.0)	23 (7.7)
II	234 (43)	215 (40)	125 (42)	98 (33)
III	92 (17)	57 (11)	103 (34)	106 (35)
IV	107 (20)	125 (23)	45 (15)	73 (24)
Missing				
Surgery type				
Breast-conserving surgery	383 (90)	368 (87)	199 (84)	181 (81)
Mastectomy	44 (10)	56 (13)	37 (16)	42 (19)
Missing	0	0	1	0
Radiation therapy				
Yes	149 (35)	150 (35)	103 (44)	90 (47)
No	278 (65)	274 (65)	130 (56)	102 (53)
Missing	0	0	6	40
Tamoxifen protocol, yrs				
1	257 (48)	261 (48)		
2	98 (18)	92 (17)		
5	186 (34)	188 (35)		
Systemic adjuvant chemotherapy				
Yes	53 (12)	42 (9.9)	203 (86)	139 (62)
No	374 (88)	382 (90)	34 (14)	84 (38)
Current ER expression				
Positive	397 (93)	411 (97)	59 (25)	56 (25)
Negative	30 (7.0)	12 (2.8)	177 (75)	165 (74)
Not available†	0.0	1 (0.2)	1 (0.4)	2 (0.9)

*The source population consisted of 11,251 female residents of the Jutland Peninsula in Denmark aged 35–69 years who were diagnosed with Stage I, II, or III breast cancer between 1985 and 2001. Subjects were estrogen receptor positive and received at least 1 year of tamoxifen therapy (ER+/TAM+) or ER negative and never received tamoxifen therapy and survived at least 1 year after diagnosis (ER-/TAM-). ApoD = Apolipoprotein D; UICC = Union for International Cancer Control.

^†^No tissue available for assay or assay results indeterminate

^§^Variable included in risk set sampling to match control subjects to case patients.

^ζ^Joint effect indicates combined nuclear and cytoplasmic dichotomous staining

Approximately half of the ER+ tumor patients were initially assigned to two-year tamoxifen treatment protocols according to the DBCG guidelines, with the remaining half split between one- and five-year protocols. Medical records often indicated a longer tamoxifen protocol compared to the registry, as patients likely were switched to the five-year protocol as evidence emerged supporting longer adjuvant treatment.[37] As expected, a much greater percentage of the ER– group was assigned to systemic chemotherapy treatment, as the overall prognosis for this subset of breast cancer patients was lower, and fewer adjuvant treatment options existed.

In both the ER+ and ER– strata, the percentage of women with some positive cytoplasmic ApoD expression was between 65% and 70%. Nuclear staining patterns differed somewhat between ER strata, with approximately 39% of ER+ tumors exhibiting positive nuclear staining, compared with 25–30% of ER– tumors (Table 1).

### Model results

In both the ER+/TAM+ and ER–/TAM– strata, all matched associations between ApoD and breast cancer recurrence were near null in both the ER+/TAM+ and ER–/TAM– strata (Table 2). For cytoplasmic staining, both dichotomous coding of ApoD staining (matched OR = 1.0, 95% CI = 0.72, 1.39), as well as continuous coding assessing dose response (matched OR = 1.0, 95% CI = 0.998, 1.002) resulted in null associations. Similarly, nuclear staining also yielded near-null results both using dichotomous coding of ApoD staining (matched OR = 1.0, 95% CI = 0.75, 1.38) and continuous coding (matched OR = 0.99, 95% CI = 0.83, 1.18). The ER–/TAM– group also yielded near null associations that were almost identical to those in the ER+/TAM+ group. The associations estimating the effect of joint nuclear and cytoplasmic expression on recurrence were also near-null in both the ER+ stratum (matched OR = 0.87, 95% CI = 0.55, 1.38) and the ER– stratum (matched OR = 1.06, 95% CI = 0.54, 2.1). Associations were similarly null among women of all stages when examined separately (S2 Table)

**Table 2:** Associations between ApoD expression and breast cancer recurrence within strata*.

ApoD Expression	ER+/TAM+			ER-/TAM-		
	Recurrent cases/controls or mean§	Matched OR (95% CI)†	Adjusted OR (95% CI)‡	Recurrent cases/controls or mean§	Matched OR (95% CI)†	Adjusted OR (95% CI)‡
Joint Expression						
= *0*	115/131			76/71		
*>0*	147/153	0.87(0.55, 1.38)	1.14 (0.79, 1.65)	65/52	1.06 (0.54, 2.10)	1.29 (0.72, 2.32)
Cytoplasmic *H-score*						
= *0*	135/144			80/77		
*>0*	292/280	1.00 (0.72,1.39)	1.19 (0.83,1.50)	157/146	0.98 (0.64,1.49)	1.14 (0.74, 1.75)
*Continuous*	Mean: 85.77/87.29	1.00 (0.996, 1.003)	1.00 (0.998,1.002)	Mean: 90.9/108.4	1.00 (0.995,1.003)	1.00 (0.996, 1.001)
Nuclear *H-score*						
= *0*	260/258			169/165		
*>0*	167/166	1.01 (0.74,1.38)	1.05 (0.78,1.40)	68/58	1.17 (0.71,1.92)	1.25 (0.79, 2.00)
*Continuous*	Mean: 1.390/1.396	1.00 (0.65,1.55)	1.03 (0.88,1.21)	Mean: 1.47/1.70	0.72 (0.38,1.37)	1.01 (0.80, 1.26)

*The source population consisted of 11,251 female residents of the Jutland Peninsula in Denmark aged 35–69 years who were diagnosed with Stage I, II, or III breast cancer between 1985 and 2001. Subjects were estrogen receptor positive and received at least 1 year of tamoxifen therapy (ER+/TAM+) or ER negative and never received tamoxifen therapy and survived at least 1 year after diagnosis (ER-/TAM-). ApoD = Apolipoprotein D; UICC = Union for International Cancer Control.

^†^Estimated using logistic regression; case patients were matched to controls on ER status, menopausal status, stage, calendar time of diagnosis, and county

^‡^Estimated using logistic regression with adjustment for time to recurrence or control selection, menopausal status, stage, receipt of chemotherapy, receipt of radiation therapy, and type of surgery.

^§^Mean for cases/controls provided for continuous variable, whereas frequency is provided for cases/controls for dichotomous exposure variable

## Discussion

In spite of plausible biological hypotheses and the results of earlier studies, we did not observe an association between tumor ApoD expression and recurrence among ER+, tamoxifen-treated patients. The effect was near null in all categories of ApoD localization—nuclear, cytoplasmic or both—and in both dichotomous and continuous categorizations of staining. Associations were near null among ER+ tumor patients and ER– tumor patients, with indiscernible differences in estimates between the two groups. This further suggests that ApoD positivity in ER+ patients is not reflective of a malfunctioning tamoxifen pathway, as any effect dependent upon the estrogen-tamoxifen pathway would be confined to the ER+ group.

This study is the largest yet investigating the association between ApoD expression and recurrence (80% power to detect a 1.5-fold change in recurrence), and resulted in precise and null estimates. Selection bias was likely avoided in the design phase, as all cases and controls were selected from the DBCG registry, which contains nearly all Danish breast cancer cases under the age of 70 at diagnosis.[33]

Tamoxifen therapy duration was often inconsistent between the DBCG registry and the patients’ medical records, with the registry indicating that the patient was assigned a shorter duration of therapy. These patients were likely initially assigned the one- or two-year protocol, and then switched to longer protocols as evidence in favor of the five-year protocol became more widespread.[37] Since patients were likely to be on tamoxifen for longer periods of time than the registry indicated, their recurrences were less likely to result from short duration therapy.

There was good concordance between ER+ status at diagnosis and upon re-assay.[39] A previous validation study using medical record review confirmed all recurrences, eliminating the potential for outcome misclassification, and showed perfect agreement for all covariates except one patient’s menopausal status.[40] Staining guidelines were designed to be clinically applicable to ensure that ApoD could be a practical predictive or prognostic indicator if an association was found.

In certain strata, such as for assessing the joint effect of nuclear and cytoplasmic staining, the sample size was fairly small. However, given the consistency of precise null results in nearly all categories of staining, it is unlikely that these estimates would change meaningfully with an increased sample size.

As with many other predictive immunohistochemical biomarkers, the threshhold expression levels to be used to discriminate between ApoD positive and negative cases remain to be established. There is insufficient information to designate such a cutoff for ApoD, but results were null when assessed using dichotomous categorization, as well as dose-response continuous coding among non-zero cores.

Earlier studies that demonstrated an association between ApoD and recurrence did so only within age-specific strata, and had smaller sample sizes.[26, 32] In order for ApoD to be prognostically relevant, it must be meaningfully associated with recurrence in largely nonspecific groups, or its stratum-specific associations must be meaningfully different. A key earlier study in particular found a potential association between ApoD and disease-specific survival in elderly, comorbid patients not receiving chemotherapy.[38] Our results are near null and similar across biologically relevant groups, suggesting that the true association between ApoD expression and recurrence is likely null. As such, the need remains for predictors of response to tamoxifen, as well as recurrence following completion of adjuvant treatment, in order to assess the need for longer duration of therapy or alternate treatments.

## Supporting information

S1 Table Bias-adjusted estimates using probabilistic methods with varying distributions. *Adjusted estimates were calculated using summary-level 2x2 tables containing exposure and outcome data using an excel spreadsheet created by Lash, Fox, and Fink^3^ †Value calculated from external validation study conducted by Soiland et al^1^ ‡Sensitivity analysis resulted in negative bias-adjusted cell values.
(PDF)

S2 Table Matched associations between ApoD expression and breast cancer recurrence within strata of ER status and stage. *The source population consisted of 11,251 female residents of the Jutland Peninsula in Denmark aged 35–69 years who were diagnosed with Stage I, II, or III breast cancer between 1985 and 2001. Subjects were estrogen receptor positive and received at least 1 year of tamoxifen therapy (ER+/TAM+) or ER negative and never received tamoxifen therapy and survived at least 1 year after diagnosis (ER-/TAM-). ApoD = Apolipoprotein D; UICC = Union for International Cancer Control. †Estimated using logistic regression; case patients were matched to controls on ER status, menopausal status, stage, calendar time of diagnosis, and county NC = Model convergence not satisfied due to sample size limitations within certain strata §Over-inflated variance due to sample size limitations within certain strata.
(PDF)

S1 File Quantitative bias analysis. (PDF)
