# Supplementary material for: Apolipoprotein D expression does not predict breast cancer recurrence among tamoxifen-treated patients
Source: PLoS One. 2017 Mar 16;12(3):e0171453. doi: 10.1371/journal.pone.0171453 (PMC5354364; doi:10.1371/journal.pone.0171453)
Supplement: S1 Table — *Adjusted estimates were calculated using summary-level 2x2 tables containing exposure and outcome data using an excel spreadsheet created by Lash, Fox, and Fink3 †Value calculated from external validation study conducted by Soiland et al1 ‡Sensitivity analysis resulted in negative bias-adjusted cell values. (PDF) [file pone.0171453.s001.pdf]

Supplementary Table S1. Bias-adjusted estimates using probabilistic methods with varying distributions

Sensitivity Trapezoidal Parameters					
**Minimum**	**Lower Mode**	**Upper Mode**	**Maximum**	**Bias-Adjusted OR (95% CI)***	**Illegal Values‡**
0.61	0.7†	0.78	0.9	1.35 (1.17-8.43)	107
0.61	0.7†	0.85	0.95	1.26 (1.14-2.26)	27
0.65	0.7†	0.85	0.95	1.26 (1.14-3.43)	30
0.65	0.75	0.85	0.95	1.24 (1.14-2.69)	14
0.7	0.75	0.85	0.95	1.22 (1.14-1.83)	0

*Adjusted estimates were calculated using summary-level 2x2 tables containing exposure and outcome data using an excel spreadsheet created by Lash, Fox, and Fink[3]

†Value calculated from external validation study conducted by Soiland et al[1]

‡Sensitivity analysis resulted in negative bias-adjusted cell values
