# Supplementary material for: Apolipoprotein D expression does not predict breast cancer recurrence among tamoxifen-treated patients
Source: PLoS One. 2017 Mar 16;12(3):e0171453. doi: 10.1371/journal.pone.0171453 (PMC5354364; doi:10.1371/journal.pone.0171453)
Supplement: S2 Table — *The source population consisted of 11,251 female residents of the Jutland Peninsula in Denmark aged 35–69 years who were diagnosed with Stage I, II, or III breast cancer between 1985 and 2001. Subjects were estrogen receptor positive and received at least 1 year of tamoxifen therapy (ER+/TAM+) or ER negative and never received tamoxifen therapy and survived at least 1 year after diagnosis (ER-/TAM-). ApoD = Apolipoprotein D; UICC = Union for International Cancer Control. †Estimated using logistic regression; case patients were matched to controls on ER status, menopausal status, stage, calendar time of diagnosis, and county NC = Model convergence not satisfied due to sample size limitations within certain strata §Over-inflated variance due to sample size limitations within certain strata. (PDF) [file pone.0171453.s002.pdf]

Supplementary Table S2. Matched Associations between ApoD expression and breast cancer recurrence within strata of ER status and stage

	Matched OR (95% CI)†					
	ER+/TAM+			ER-/TAM-		
**ApoD Expression**	**Stage I**	**Stage II**	**Stage III**	**Stage I**	**Stage II**	**Stage III**
Joint Expression						
=0						
>0	NC	1.08 (0.69-1.70)	0.86 (0.55-1.34)	1.00 (0.06-15.99) §	1.11 (0.59-2.10)	1.46 (0.68-3.13)
Cytoplasmic *H-score*						
=0						
>0	1.00 (0.06-15.99) §	1.19 (0.71-1.98)	0.88 (0.57-1.37)	1.00 (0.14-7.10)	1.04 (0.60-1.82)	0.88 (0.44-1.77)
Continuous	1.016 (0.97-1.07)	0.995 (0.99-1.00)	1.00 (0.998-1.01)	NC	0.999 (0.992-1.004)	0.998 (0.993-1.003)
Nuclear *H-score*						
=0						
>0	NC	1.20 (0.77-1.88)	0.91 (0.59-1.40)	NC	1.06 (0.55-2.01)	1.27 (0.58-2.80)
Continuous	NC	0.97 (0.52-1.83)	1.01 (0.55-1.85)	NC	0.69 (0.30-1.58)	0.65 (0.20-2.14)

The source population consisted of 11,251 female residents of the Jutland Peninsula in Denmark aged 35-69 years who were diagnosed with tage I, II, or III breast cancer between 1985 and 2001. Subjects were estrogen receptor positive and received at least 1 year of tamoxifen therapy ER+/TAM+) or ER negative and never received tamoxifen therapy and survived at least 1 year after diagnosis (ER-/TAM-).

poD=Apolipoprotein D; UICC=Union for International Cancer Control.

Estimated using logistic regression; case patients were matched to controls on ER status, menopausal status, stage, calendar time of diagnosis, nd county

C=Model convergence not satisfied due to sample size limitations within certain strata

Over-inflated variance due to sample size limitations within certain strata
