# Supplementary material for: Apolipoprotein D expression does not predict breast cancer recurrence among tamoxifen-treated patients
Source: PLoS One. 2017 Mar 16;12(3):e0171453. doi: 10.1371/journal.pone.0171453 (PMC5354364; doi:10.1371/journal.pone.0171453)
Supplement: S1 File — (PDF) [file pone.0171453.s003.pdf]

Supplementary File S1. Quantitative Bias Analysis

Apo-D Validation

One major drawback to the use of tissue microarrays in this study was its potential to be incompletely representative of the heterogeneity of certain tumors.[1] This heterogeneity is not apparent in the results, leading to potential exposure misclassification that is likely to be non-differential, as scorers were blinded to disease status.

Data from a paper by Soiland et al were used to determine parameters for sensitivity and specificity[1]. In their study, TMAs were sampled only from the invasive front, and produced a sensitivity of 70% and perfect specificity when compared with whole section staining. In our study, since four TMAs were sampled from different parts of the tumor to obtain representative staining, the sensitivity was likely to be higher. Several trapezoidal distributions were applied assigned to the sensitivity to account for the likely under-estimation of sensitivity in the external validation data with respect to our study.

Positive and negative predictive values were calculated from these sensitivity values, and incorporated into a probabilistic bias analysis. Using Monte Carlo simulation, these values and their distributions were applied to summary-level data stratified on menopausal status using an excel spreadsheet developed by Lash, Fox, and Fink in order to obtain reclassified counts for positive (>0) and negative (=0) H-scores, and bias-adjusted measures of association with a 95% simulation interval [2,3].

Bias-Adjusted Results

Bias-adjusted estimates were slightly further from the null than conventional estimates on average among ER+ women (Bias-Adjusted OR = 1.35, SI = 1.17-6.6), as well as ER- women (Bias-Adjusted OR = 1.1, SI = 1.05-2.6). Even after taking the potential for exposure misclassification into account, the association between ApoD expression and recurrence appears to be weak or null and non-differential across ER strata, suggesting that ApoD plays a minimal role in recurrence risk via the tamoxifen-ER pathway.

When calculated probabilistically, bias-adjusted estimates varied depending on the trapezoidal parameters selected (Supplementary Table S1). The largest bias-adjusted OR was calculated using a lower range of sensitivities, and suggests that ApoD may have a weak effect on recurrence (Bias-adjusted OR=1.35, 95%CI: 1.17-8.43). A trapezoidal distribution using the upper range of sensitivity parameters resulted in a weaker association (Bias-adjusted OR=1.22, 95% CI=1.14-1.83).

References

1. Soiland, H., Skaland, I., Janssen, E. A., Gudlaugsson, E., Korner, H., Varhaug, J. E., Baak, J. P. (2008). Comparison of apolipoprotein D determination methods in breast cancer. *Anticancer Res, 28*(2B), 1151-1160.
2. Fox MP, Lash TL, Greenland S. A method to automate probabilistic sensitivity analyses of misclassified binary variables. International Journal of Epidemiology 2005.
3. Fox MP, Lash TL, Greenland S. Sensitivity Analysis Macro. 2009. <https://sites.google.com/site/biasanalysis/sensmac>
